# P013. 25(OH)D Level and headache in children sample

**DOI:** 10.1186/1129-2377-16-S1-A84

**Published:** 2015-09-28

**Authors:** Elisabetta Tozzi, Alessandra Boncristiano, Annarita Antenucci, Simona Di Loreto, Giovanni Farello

**Affiliations:** Neuropsychiatric Clinic, Hospital of L'Aquila, Department of life, health and environmental sciences, University L'Aquila, L'Aquila, Italy; Pediatric Clinic, Hospital of L'Aquila, L'Aquila, Italy

## Background

Statistical significance between serum 25(OH)D levels and headache in the pediatric population is still controversial. However, a supplementary vitamin D therapy in children with headache allowed us to note an improvement in the clinical symptoms in terms of frequency and severity[1].

## Aim

To study vitamin D blood levels in primary headaches and to show the relationship with clinical parameters influencing clinical course of the headache.

## Materials and methods

Sixty-seven males (42%) and 92 females (58%), aged between 5 to 18 years, suffering from headache, were divided into three diagnostic categories, according to the ICHD-III classification: migraine with aura (MWA), migraine without aura (MWoA) and tension-type headache (TTH). Serum vitamin D level lower than 20 ng/ml was considered pathological. Immunohistochemical methods of chemiluminescence were used to determine blood 25(OH)D level.

## Results

Ninety-one patients (57%) received a diagnosis of MWoA, 32 (20%) of MWA and 36 (23%) of TTH. Hypovitaminosis D was found in 56% of the children with MWoA, in 50% of patients with MWA and in 44.4% with TTH. Twelve percent of all the sample showed severe Hypovitaminosis D (<10 ng/ml). There were neither statistically significant differences comparing vitamin D serum levels between males and females in each category of headache (p = 0.36), nor more severe deficiency of vitamin D in overweight children with BMI>90°C (p = 0.47), and serum concentration was not lower in adolescents and pre-adolescent than children under 10 years. A difference resulted in the limits of statistical significance (p = 0.07) in 25(OH)D serum level in children with MWoA compared to patients with MWA and THH.

## Conclusions

Our study shows a high incidence of vitamin D deficiency in the pediatric population with headache (56%), particularly in migraineurs without aura[2]. Probably this deficiency could be directly correlated with a higher frequency of migraine attacks in children with MWoA.

Written informed consent to publication was obtained from the patient(s).
